# Body weight dissatisfaction by socioeconomic status among obese, preobese and normal weight women and men: results of the cross-sectional KORA Augsburg S4 population survey

**DOI:** 10.1186/1471-2458-12-342

**Published:** 2012-05-09

**Authors:** Thomas von Lengerke, Andreas Mielck

**Affiliations:** 1 Medical Psychology Unit (OE 5430), Hannover Medical School, Carl-Neuberg-Str. 1, 30625, Hannover, Germany; 2 Institute of Health Economics and Health Care Management, Helmholtz Center Munich – German Research Center for Environmental Health, Ingolstädter Landstraße 1, 85764, Neuherberg, Germany

**Keywords:** Obesity, Preobesity, Normal weight, Body weight dissatisfaction, Socioeconomic status, Gender

## Abstract

**Background:**

Body weight dissatisfaction is an important factor in preventing weight gain and promoting weight loss or maintenance. This study focuses on differences in the rates of body weight dissatisfaction among obese, preobese and normal weight women and men by socioeconomic status within a general adult population in Germany.

**Methods:**

Data were analyzed from 4186 adults aged 25 to 74 who participated in a cross-sectional, representative population-based health survey (KORA S4, 1999–2001, Augsburg region/Germany). Body mass was measured anthropometrically and indexed following international standards. Among the 2123 women participating in the survey, 40.3% had a normal weight, 34.9% were preobese, and 24.8% were obese (compared to 25.9%, 51.4% and 22.6% among men, respectively). Body weight dissatisfaction, educational level, household income and occupational status were assessed by computer-aided personal interviewing. An index for socioeconomic status was calculated and categorized into quintiles. Multiple logistic regressions were performed to test for differences in the odds of body weight dissatisfaction across socioeconomic strata in normal weight, preobese and obese groups. Body mass index, age, family status, place of residence and health behaviors were adjusted for.

**Results:**

Overall, being dissatisfied with one’s body weight was more prevalent in women (48.3%) than in men (33.2%). In the normal weight group, no significant differences in the odds of being dissatisfied were found across socioeconomic groups among women or men. Among preobese men, compared to the lowest socioeconomic stratum, increased odds of being dissatisfied with one’s body weight were associated with the highest socioeconomic index group (OR = 2.3, 95% CI: 1.4–3.8), middle and high educational level (OR = 1.6, 95% CI: 1.1–2.3, and OR = 1.9, 95% CI: 1.3–3.7), high income (OR = 1.8, 95% CI: 1.2–2.7), and middle and high occupational status (both OR = 1.8, 95% CI: 1.2–2.6). Among preobese women, the odds of being dissatisfied were only significantly elevated in those with a middle educational level (OR = 1.6, 95% CI: 1.1–2.3). Among obese men, elevated odds were found in the highest socioeconomic index group (OR = 3.7, 95% CI: 1.8–7.5) and in those with a high educational level (OR = 2.3, 95% CI: 1.3–4.1), high income (OR = 2.6, 95% CI: 1.4–4.7), and middle and high occupational status (both OR = 2.2, 95% CI: 1.3–3.6). The odds of dissatisfaction among obese women were not associated with socioeconomic status as a whole, but were associated with a high educational level, albeit with a comparatively large confidence interval (OR = 3.6, 95% CI: 1.0–12.8).

**Conclusions:**

In Germany, body weight dissatisfaction is more prevalent among obese and preobese men in high socioeconomic status groups, a pattern not found in women. The exception to this is a greater prevalence of dissatisfaction among obese and preobese women with a high educational level (albeit inconsistently). Moreover, there is a social gradient in body weight dissatisfaction, especially in obese men, which may partly explain why obesity is more prevalent in men with low socioeconomic status. It also suggests that they are a target group for obesity care in which body weight satisfaction is an important topic.

## Background

Body image and, more specifically, body weight dissatisfaction have been shown to be positively associated with intending or trying to lose weight in adults [1-5]. Obviously, this does not guarantee the eventual success of such intentions or attempts. Quite the contrary, there is at least mixed evidence concerning the assumption that body weight dissatisfaction is a pre-treatment barrier to weight control [6,7]. However, this evidence only pertains to patients already enrolled in an obesity treatment program. Thus, it has been argued that being dissatisfied with one’s body weight may not only be a risk factor for eating disorders and obesity (such as in adolescents [8] or chronic dieters [9]). It may also be a potential motivator for initiating healthy lifestyle changes [7], especially among obese adults [10], as long as it does not exceed critical levels which may actually impede motivation [11].

In any case, there is little doubt that body weight dissatisfaction is an important factor in preventing weight gain and promoting weight loss or maintenance. The distribution of body weight dissatisfaction among relevant target groups is, therefore, of particular interest. Within economically wealthy countries with Western lifestyles, these groups include those with relatively low socioeconomic status (SES) which tend to be affected by the highest obesity rates [12]. In these countries where body dissatisfaction is most common (most notably in the US [13,14]), it has been asserted that women—particularly those with a comparatively high SES—are those most dissatisfied with their body and body weight [15]. For men, such research has traditionally been more scarce [16]. Studies from some European countries have shown, however, that higher SES is associated with higher odds of body weight dissatisfaction in men, but not women [17,18].

Most studies on the relationship between SES and body weight dissatisfaction have tended to scrutinize this association in general, rather than looking at normal weight, preobese or obese subgroups separately [1,3-5,15,17,18]. A similar assertion holds for self-perceived weight appropriateness [19-21], a construct related to though distinct from body weight dissatisfaction [22]. Hypothetically, the positive association between SES and body weight dissatisfaction should primarily be present among overweight groups, given that those with high SES “…have more narrowly defined standards for acceptable body size” [20]. In other words, although those in the normal weight range must also meet relatively narrow standards, roughly increased odds of body weight dissatisfaction in high SES groups should be found for those who are overweight since they more strongly deviate from the defined standards. Alternatively, if the assertion holds that “…social class is not related to body shape ideals, since people from different social classes present similar ideals” [23], social inequalities in body weight dissatisfaction should be rather small, regardless of body weight status.

Against this background, this study presents further analyses of German population-based data from the KORA S4 study [18]. In specific, it examines the distribution of body weight dissatisfaction across different socioeconomic status groups among normal weight, preobese and obese women and men.

## Methods

### Population and sampling

Data for the study were taken from the KORA S4 Survey 1999/2001, a representative cross-sectional health survey conducted in the city of Augsburg, Germany, and its two adjacent rural administrative districts (KORA: Cooperative Health Research in the Region of Augsburg [24]). Approval for the survey was obtained from the responsible ethics committee (Bavarian Medical Association, Munich). The target population consisted of German residents born between July 1, 1925 and June 30, 1975. A sample of 6640 residents was drawn using a two-stage sampling procedure. First, in addition to the city of Augsburg, 16 out of 70 communities from were chosen from the adjacent districts by cluster sampling with probability proportional to size. Using public registry office listings, stratified random sampling was then performed within each community, yielding ten strata of equal size based on gender and age. (For selection within strata, the RANUNI function in SAS 8.1 for Windows was used). Fieldwork lasted from October 1999 to April 2001. N = 4261 residents participated in the survey, yielding a response rate of 67%, which is good compared to other surveys [25]. N = 29 residents for whom no body mass index could be determined due to missing data, N = 26 who were underweight and N = 20 with missing SES data were excluded. Thus, N = 4186 were available for analysis.

A telephone survey of non-responders (response rate: 49%) revealed that they more often had a low level of education (German *Grundschule* or *Hauptschule*: 65% vs. 54%) and fair or poor self-rated health (28% vs. 21%) compared to those who participated. Non-responders were also more often unmarried (34% vs. 29%) and smokers (29% vs. 26%) and more frequently reported physician contact within the previous four weeks (46% vs. 38%), myocardial infarction (6% vs. 3%), and diabetes (7% vs. 4%) [25].

### Measures

#### Obesity

Body weight and height were measured anthropometrically following international standards during the physical examination part of the survey [24,26]. Participants stood without shoes and heavy outer garments [27], and column scales (SECA 709) with integrated measuring rods (SECA 221) were used. Calibration of instruments was ensured by carrying out weekly or daily inspections using standard weights or resistors. Body mass index (BMI) was calculated by dividing weight in kg by height in m². BMI groups were defined based on the WHO classification [28]: normal weight (18.5 ≤ BMI < 25), preobesity (25 ≤ BMI < 30), and obesity (BMI ≥ 30). In addition, key analyses were run for participants with moderate obesity (30 ≤ BMI < 35) in order to assess the impact of severe obesity (BMI ≥ 35) on the association between SES and body weight dissatisfaction among obese groups. (Separate analyses for severe obesity were not possible due to small subsample sizes.)

#### Socioeconomic status (SES)

Educational level, income and occupational status were assessed via computer-aided personal interviewing following national recommendations [29] and later aggregated into an SES index using an algorithm often used in Germany [30]. Variables were defined as highest educational level, net equivalized household income (relative to number and age of household members with weights of 1 for the head of the household, 0.5 for household members aged ≤ 6 years, 0.65 for those aged 7–14, 0.9 for those aged 15–17, and 0.8 for those 18 or older), and current or former occupational status (own or partner’s). The SES index makes it possible to categorize individuals into five groups (quintiles) ranging from lowest to highest SES. For additional analyses, these five groups were merged into three groups (low, middle, high).

In the analyses presented below, the SES indicators were also analyzed separately. Educational level was classified as low (German *Grundschule* or *Hauptschule*, i.e., lower secondary school or less), middle (*Realschule*, i.e., intermediate secondary school) or high (*Gymnasium*, i.e., upper secondary school, i.e., *Gymnasium*), representing the basic German school system. To facilitate comparisons across SES indicators, income and occupational status were also trichotomized, with income being grouped into terciles and occupational status being categorized into low (un-/semi-skilled workers or employees, civil servants and skilled workers with simple tasks), middle (foremen and master craftsmen, employees and civil servants with moderate tasks) and high (self-employed persons or employees and civil servants with higher/executive functions).

#### Body weight dissatisfaction

Body weight dissatisfaction was assessed via computer-aided personal interviewing and operationalized with the item “How satisfied are you with your body weight?” (1: very satisfied, 2: rather satisfied, 3: rather dissatisfied, 4: very dissatisfied). For the present study, this item was dichotomized into satisfied (“very satisfied” or “rather satisfied”) and dissatisfied in order to keep presentation of the models as ostensive as possible and to avoid jeopardizing their robustness due to small subsample sizes and low frequencies of item values (especially endpoints) in strata by sex, BMI and SES (e.g., “very satisfied” obese participants in higher SES groups) [18]. However, key analyses were also conducted with the original 4-point scale to determine whether the results would be consistent with those obtained though use of the dichotomized variable.

#### Covariates

Data on sex, age and place of residence (urban vs. rural) were obtained through the sampling procedure (public registry office listings; see above). Family status and health-related behaviors were self-reported via computer-aided personal interviewing. Smoking was assessed by the item “Do you currently smoke cigarettes?” followed by “Do you smoke regularly or occasionally (i.e. on average less than one cigarette a day)?” for smokers and by “Have you ever smoked cigarettes?” for non-smokers. Based on their responses to these questions, participants were categorized as smokers (incl. occasional smokers), ex-smokers and never smokers. Alcohol consumption was measured using a recall method in which the intake of beer, wine and spirits on the weekend and weekday prior to interview was extrapolated to the full previous week. Average intake was then dichotomized based on toxic thresholds (men: > 40 g/day, women: > 20 g/day [31]). Smoking was adjusted for in the analyses since it may adversely affect body image concerns among women [32], and alcohol consumption was adjusted for since it has been shown to diminish stress responses associated with a body image-related social stressor among women [33]. Physical activity was assessed by two items asking participants how often they exercise during the summer and winter. Participants were then categorized as very active (> 2 hours of regular exercise per week during both seasons), moderately active (≥ 1–2 hours of regular exercise per week during both seasons, or > 1 hour per week during one season but > 2 hours per week during the other), somewhat active (≥ 1–2 hours of regular exercise per week during one season) and inactive (> 1 hour of exercise per week during both seasons). Nutritional behavior was operationalized using a food intake frequency instrument [34] allowing classification of participants as has having optimal, moderate or unfavorable nutritional behavior according to German Nutrition Society guidelines. Weight reduction dieting was assessed with the item “In the last 12 months, have you dieted in order to lose weight?”

#### Statistical analysis

IBM SPSS Statistics version 19 was used for the statistical analyses. First, SES was cross-tabulated with BMI group, age, family status, place of residence and body weight dissatisfaction in order to describe the total sample and SES subgroups. Second, the rates of body weight dissatisfaction within every BMI x SES subgroup were calculated without adjustment for covariates (bivariate analysis). Third, multiple logistic regression analyses were conducted to test for differences in the rates of body weight dissatisfaction across SES groups within the obese, preobese, and normal weight subgroups, respectively. One model for moderately obese women and one for moderately obese men were also run using the SES index in order to evaluate the influence of severe obesity on the association between SES and body weight dissatisfaction. In addition, in order to check for possible effects of dichotomization of the dissatisfaction item on results, differences in the results obtained across SES index groups using the original 4-point scale were analyzed using general linear modeling (UNIANOVA). In each model, the lowest SES group was defined as the reference group, and BMI, age, family status, place of residence and a number of health-related behaviors were adjusted for. All analyses were conducted for women and men separately. No outlier trimming was performed.

## Results

### Sample description

Table 1 describes the sample which consisted of 2123 women and 2063 men. In total, more women (40.3%) than men (25.9%) had a normal weight, while proportions of obese respondents were rather similar across sexes (women: 24.8%, men: 22.6%). In lower SES groups, obesity rates exceeded those in higher social echelons, especially among women (11.9% in the highest SES group; up to 38.9% in the lowest group). An analogous trend was found for age, again particularly among women. The proportion of aged respondents was higher (31.3%) among women with the lowest SES. Women were more often divorced or widowed (16.5% versus 9.2% for men), once again predominantly among lower SES subgroups (lowest SES: 23.3% of women vs. 9.4% of men). By contrast, place of residence was essentially distributed equally across socioeconomic groups, both among women and men. Finally, in these unadjusted bivariate analyses, proportions of body weight dissatisfaction—which were generally higher in women (48.3%) than in men (33.2%)—declined with SES among women (from 56.2% in the highest SES group to 38% in the lowest), while they tended to increase with SES among men.

**Table 1:** **Sample description**^1,2^

	**1:Lowest SES**^**3**^		**2**		**3**		**4**		**5:Highest SES**		**Total**	
**Women**	**N = 550**		**N = 374**		**N = 538**		**N = 400**		**N = 261**		**N = 2123**	
**Normal weight**^4^	127	23.1%	143	38.2%	227	42.2%	196	49.0%	163	62.5%	856	40.3%
**Preobese**	209	38.0%	133	35.6%	195	36.2%	136	34.0%	67	25.7%	740	34.9%
**Obese**	214	38.9%	98	26.2%	116	21.6%	68	17.0%	31	11.9%	527	24.8%
**25-35 years of age**	49	8.9%	65	17.4%	123	22.9%	99	24.8%	80	30.7%	416	19.6%
**35-45 years of age**	81	14.7%	82	21.9%	116	21.6%	108	27.0%	64	24.5%	451	21.1%
**45-55 years of age**	101	18.4%	75	20.1%	123	22.9%	95	23.8%	61	23.4%	455	21.4%
**55-65 years of age**	147	26.7%	92	24.6%	102	19.0%	65	16.3%	29	11.1%	435	20.5%
**65-75 years of age**	172	31.3%	60	16.0%	74	13.8%	33	8.3%	27	10.3%	366	17.2%
**Married**	395	71.8%	266	71.1%	376	69.9%	286	71.5%	150	57.5%	1473	69.4%
**Single**	27	4.9%	36	9.6%	87	16.2%	70	17.5%	80	30.7%	300	14.1%
**Divorced/widowed**	128	23.3%	72	19.3%	75	13.9%	44	11.0%	31	11.9%	350	16.5%
**Urban place of residence**	224	40.7%	153	40.9%	239	44.4%	199	49.8%	126	48.3%	941	44.3%
**Rural place of residence**	326	59.3%	221	59.1%	299	55.6%	201	50.2%	135	51.7%	1182	55.7%
**Body weight satisfaction**	235	43.8%	188	51.1%	278	53.0%	216	54.8%	160	61.8%	1077	51.7%
**Body weight dissatisfaction**	302	56.2%	180	48.9%	247	47.0%	178	45.2%	99	38.2%	1006	48.3%

**MEN**	**N = 373**		**N = 394**		**N = 382**		**N = 435**		**N = 479**		**N = 2063**	
**Normal weight**	86	23.1%	94	23.9%	95	24.9%	119	27.4%	141	29.4%	535	25.9%
**Preobese**	173	46.4%	207	52.5%	193	50.5%	228	52.4%	260	54.3%	1061	51.4%
**Obese**	114	30.6%	93	23.6%	94	24.6%	88	20.2%	78	16.3%	467	22.6%
**25**–**35 years of age**	65	17.4%	70	17.8%	72	18.8%	96	22.1%	100	20.9%	403	19.5%
**35**–**45 years of age**	70	18.8%	65	16.5%	79	20.7%	99	22.8%	103	21.5%	416	20.2%
**45**–**55 years of age**	56	15.0%	75	19.0%	82	21.5%	92	21.1%	113	23.6%	418	20.3%
**55**–**65 years of age**	83	22.3%	92	23.4%	74	19.4%	92	21.1%	95	19.8%	436	21.1%
**65**–**75 years of age**	99	26.5%	92	23.4%	75	19.6%	56	12.9%	68	14.2%	390	18.9%
**Married**	289	77.5%	274	69.5%	295	77.2%	302	69.4%	326	68.1%	1486	72.0%
**Single**	49	13.1%	74	18.8%	67	17.5%	83	19.1%	115	24.0%	388	18.8%
**Divorced/widowed**	35	9.4%	46	11.7%	20	5.2%	50	11.5%	38	7.9%	189	9.2%
**Urban place of residence**	150	40.2%	190	48.2%	169	44.2%	198	45.5%	242	50.5%	949	46.0%
**Rural place of residence**	223	59.8%	204	51.8%	213	55.8%	237	54.5%	237	49.5%	1114	54.0%
**Body weight satisfaction**	255	71.6%	269	70.1%	247	65.7%	275	64.4%	301	63.8%	1347	66.8%
**Body weight dissatisfaction**	101	28.4%	115	29.9%	129	34.3%	152	35.6%	171	36.2%	668	33.2%

*Notes:*^1^Sex and age were used as stratification dimensions during sampling (for details, see text); ^2^table shows column %; ^3^SES: socioeconomic status; ^4^normal weight: 18.5 ≤ BMI < 25; preobese: 25 ≤ BMI < 30; obese: BMI ≥ 30.

### Bivariate analysis of body weight dissatisfaction by SES for different BMI groups

**Figure 1:**
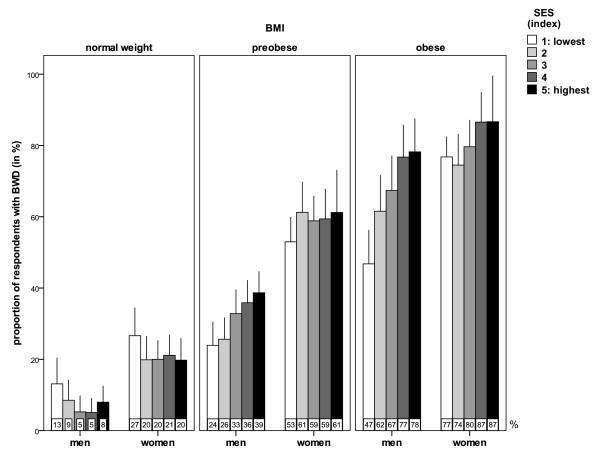
Body weight dissatisfaction (BWD) by BMI status and SES index.

Figure 1 shows the proportions of respondents with body weight dissatisfaction in different SES groups defined by the SES index. Differences varied both by BMI and gender. Among normal weight women and men, dissatisfaction was most prevalent among those with the lowest SES; only small differences were found across the other SES groups. In the preobese group, whereas the odds of being dissatisfied increased with increasing SES among men, this finding was less clear and pronounced in women. These trends appear to be the same in the obese groups. Large differences in body weight dissatisfaction rates were found across SES groups in obese men, with a particularly low rate of dissatisfaction in the lowest SES group. Differences were smaller among obese women, among whom the difference in rates between the highest and the lowest SES group was 10%. By contrast, this difference was 31% among obese men. Among moderately obese participants, rates of being dissatisfied in the five SES groups were 43%, 61%, 61%, 77% and 79% among men and 73%, 70%, 81%, 84% and 85% among women, respectively (not shown).

**Figure 2:**
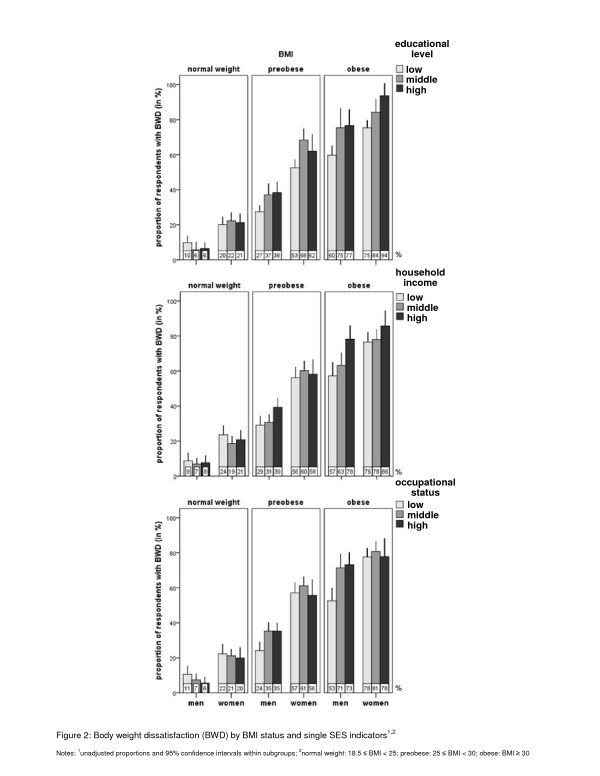
Body weight dissatisfaction (BWD) by BMI status and single SES indicators.

Figure 2 shows the corresponding analyses for the single SES indicators (i.e., educational level, household income, and occupational status). For men, results by and large compare well with those shown in Figure 1. There were slight variations in body weight dissatisfaction rates across socioeconomic groups in the normal weight group. These variations increased in the overweight groups, with the most pronounced differences being found in the obese group. Among obese men, differences in dissatisfaction rates between the high and low SES groups in terms of educational level, income and occupational status were 17%, 21% and 20% respectively. For women, results again largely resemble those found in the analyses with the SES index, with the only exception being that certain greater differences in dissatisfaction rates were found this time with respect to educational level. Among preobese women, the likelihood of being dissatisfied was highest for those with a middle educational level (15% higher than for those with the lowest educational level), and among obese women, the likelihood was greatest for those with the highest level of education (19%).

### Multiple logistic regression models of body weight dissatisfaction by SES for different BMI groups

Table 2 shows the results of the logistic models analyzing the odds of body weight dissatisfaction in subgroups defined by the SES index, adjusting for BMI, age, family status, place of residence, and health-related behaviors. Among normal weight women and men, none of the differences found in the unadjusted analysis (Figure 1) were statistically significant. In the preobese group, the odds of being dissatisfied were 1.9 and 2.4 times higher for men in SES groups 4 and 5 (highest SES), respectively, than in group 1 (lowest SES), while no significant differences were found in preobese women. The same pattern recurs in the obese group, albeit with larger odds ratios for men: odds of dissatisfaction in groups 3, 4 and 5 (highest SES) were 2.1, 3.3 and 3.7 times higher than in group 1 (lowest SES). It is also important to note that the odds for preobese or obese men increase with SES in a dose–response manner (p > .001 for linear trends using polynomial contrasts). This also holds when analyzing moderately obese men separately, among whom the odds of be dissatisfied were two times higher in SES groups 2 and 3, 4.4 times higher in group 4, and 5.5 times higher in group 5 (all p < .05) than in the lowest SES group (not shown).

**Table 2:** **Odds of body weight dissatisfaction by SES: Results of logistic regression models using the SES index**^1^

	**Normal**^**2**^				**Preobese**				**Obese**			
	**Men**		**Women**		**Men**		**Women**		**Men**		**Women**	
	**OR**^**3**^	**95% CI**	**OR**	**95% CI**	**OR**	**95% CI**	**OR**	**95% CI**	**OR**	**95% CI**	**OR**	**95% CI**
**5: Highest SES**^4^	0.8	0.3–2.2	0.7	0.4–1.3	**2.4**	**1.5**–**3.9**	1.3	0.7–1.8	**3.7**	**1.8**–**7.5**	1.5	0.5–4.8
**4**	0.4	0.1–1.3	0.7	0.4–1.2	**1.9**	**1.5**–**3.9**	1.1	0.7–1.6	**3.3**	**1.7**–**6.3**	1.5	0.7–3.6
**3**	0.4	0.1–1.4	0.8	0.4–1.4	1.5	0.9–2.5	1.0	0.7–1.8	**2.1**	**1.1**–**3.8**	0.9	0.5–1.7
**2**	0.8	0.3–2.2	0.8	0.4–1.4	1.2	0.7–1.9	1.1	0.7–2.5	1.6	0.8–2.9	0.8	0.5–1.5
**1: Lowest SES**	1.0	-	1	-1.9	1.0	-	1	-1	1	-	1	-

*Notes:*^1^Adjusted for BMI, age, family status, place of residence, smoking, alcohol consumption, physical activity, nutritional behavior, and weight reduction dieting;

^2^normal weight: 18.5 ≤ BMI < 25; preobese: 25 ≤ BMI < 30; obese: BMI ≥ 30; ^3^odds ratios in bold indicate p < .05; ^4^*SES*: socioeconomic status.

General linear modeling of the 4-point scale of body weight dissatisfaction revealed that for women, results were similar to those of the logistic regressions, with no significant differences occurring across the five SES categories in the normal weight group (F(4,827) = 0.16, *p* = .96), the preobese group (F(4,797) = 0.99, *p* = .411) or the obese group (F(4,504) = 0.46, *p* = .761). Results for the men also reflected those of the logistic models in that there were significant differences in mean body weight dissatisfaction across SES groups in the obese (F(4,442) = 3.87, *p* = .004) and preobese groups (F(4,1015) = 7.49, *p* < .001), but not in the normal weight group (F(4,515) = 1.8, *p* = .127). Among preobese men, significant contrasts to the lowest SES group (mean = 2) were found for SES groups 3 (mean = 2.1, *p* = .029), 4 (mean = 2.2, p = .001) and 5 (mean = 2.3, *p* < .001), and among obese men (mean of the lowest SES group: 2.5) for groups 2 (mean = 2.7, *p* = .034), 3 (mean = 2.7, *p* = .013), 4 (mean = 2.8, *p* < .001) and 5 (mean = 2.8, *p* = .003).

Finally, Table 3 shows the logistic regression models with the single SES indicators and, for better comparison, the three-level version of the SES index. Again, no significant differences were found among normal weight participants. Preobese men with a middle or high educational level, high income, and middle or high occupational status show significantly increased odds of dissatisfaction compared to the respective low SES level. A similar picture can be observed for obese men, namely increased odds given a high educational level, high income, and middle and high occupational status. In addition, practically all odds ratios are larger than in the preobese group. Most associations reveal the same dose–response pattern described in Table 1. Looking at the three-level SES index, dissatisfaction odds in the case of low SES are greater than for low educational level, low income or low occupational status, which indicates that the SES index may capture these social inequalities better than the single SES indicators.

**Table 3:** **Odds of body weight dissatisfaction by SES: Results of logistic regression models using single SES indicators**^1^

	**Normal**^**2**^				**Preobese**				**Obese**			
	**Men**		**Women**		**Men**		**Women**		**Men**		**Women**	
	**OR**^**3**^	**95% CI**	**OR**	**95% CI**	**OR**	**95% CI**	**OR**	**95% CI**	**OR**	**95% CI**	**OR**	**95% CI**
***SES***^**4**^***(index)***												
**High**	0.8	0.3–2.2	0.7	0.4–1.2	**2.3**	**1.4**–**3.8**	1.3	0.7–2.5	**3.7**	**1.8**–**7.5**	1.5	0.4–4.8
**Middle**	0.5	0.2–1.3	0.8	0.4–1.4	1.5	0.9–2.3	1.1	0.7–1.6	**2.1**	**1.3**–**3.5**	1.0	0.6–1.6
**Low**	1	-	1	-	1	-	1	-	1	-	1	-
***Educational level***												
**High**	0.7	0.3–1.6	1.2	0.7–1.9	**1.9**	**1.3**–**3.7**	1.4	0.8–2.2	**2.3**	**1.3**–**4.1**	***3.6***	***1.0***–***12.8***
**Middle**	0.6	0.2–1.7	1.1	0.7–1.7	**1.6**	**1.1**–**2.3**	**1.6**	**1.1**–**2.3**	1.8	0.9–3.5	1.5	0.8–3.0
**Low**	1	-	1	-	1	-	1	-	1	-	1	-
***Household income***												
**High**	1.1	0.5–2.8	0.9	0.6–1.5	**1.8**	**1.2**–**2.7**	1.3	0.8–2.2	**2.6**	**1.4**–**4.7**	1.8	0.8–4.2
**Middle**	0.9	0.4–2.1	0.7	0.5–1.2	1.3	0.9–1.8	1.5	0.9–2.1	1.2	0.8–1.9	1.0	0.6–1.7
**Low**	1	-	1	-	1	-	1	-	1	-	1	-
***Occupational status***												
**High**	0.6	0.4–1.8	0.9	0.5–1.6	**1.8**	**1.2**–**2.6**	0.7	0.4–1.2	**2.2**	**1.3**–**3.6**	0.9	0.4–1.8
**Middle**	0.8	0.2–1.4	0.9	0.6–1.4	**1.8**	**1.2**–**2.6**	1.1	0.8–1.6	**2.2**	**1.3**–**3.6**	0.9	0.5–1.5
**Low**	1	-	1	-	1	-	1	-	1	-	1	-

Notes: ^1^Adjusted for BMI, age, family status, place of residence, smoking, alcohol consumption, physical activity, nutritional behavior, and weight reduction dieting;

^2^normal weight: 18.5 ≤ BMI < 25; preobese: 25 ≤ BMI < 30; obese: BMI ≥ 30; ^3^odds ratios in bold indicate p < .05, those in bold italics indicate *p* = .05;

^4^*SES*: socioeconomic status.

Among women, significant differences were found for educational level, especially in the subgroup of obese women. Those with a high educational level were 3.6 times more likely to be dissatisfied with their weight than those with a low educational level, albeit with a rather large confidence interval and marginal statistical significance (p = .05).

## Discussion

To summarize the results of the present study, no significant differences in the odds of body weight dissatisfaction were found for normal weight women and men by SES. Among the preobese, increased odds of body weight dissatisfaction were found in men with the highest SES index values, a middle or high educational level, high income, and middle or higher occupational status, while in women, odds were only increased for those with a middle educational level. In the obese groups, SES differences in body weight dissatisfaction were greater than in the non-obese groups. Among men, odds were up to 3.7 times higher in the group with the highest SES. As in the group of preobese men—though even more pronounced—a dose–response gradient in dissatisfaction was observed across SES groups. Among obese women, however, again no significant differences were found except that those with a high educational level had marginally significant higher odds of dissatisfaction than those with the lowest level of education. The analyses conducted for moderately obese participants revealed results that were essentially similar to those for the obese groups as a whole.

Among the strengths of the present study is that its data come from a survey which used rigorous quality assurance procedures [24]. Body weight and height were measured (not self-reported), and sample size allowed for stratification for both BMI status and gender. At the same time, the study also has a number of noteworthy limitations. First, body weight dissatisfaction was assessed by a single item only. The main reason for this was practicality since the survey from which data were taken was an omnibus survey which already took participants an average of three hours to complete. While comparably simple items have been used in previous epidemiological studies [3,5], the use of a single item for measuring body weight dissatisfaction implies that the reliability of this indicator is uncertain and that there is a need for replication using a more sophisticated measure (e.g., the Body Areas Satisfaction Scale [35]). Regarding the sensitivity of this single dichotomized item, cross-checks of the logistic models with linear models using the original 4-point scale indicated that the results essentially lead to similar conclusions, with the only exception being that mean differences on the scale appear to be of less magnitude than differences in percentages of respondents being dissatisfied. A second limitation is that the survey was regionally confined. Since body weight dissatisfaction may vary systematically with regional affluence and obesity prevalence [36,37], studies conducted in other regions or at the national level controlling for regional attributes could clarify whether similar patterns would emerge under different circumstances. Third, no measure of perception regarding weight status as such was available. In other words, lack of awareness of overweight status among men with lower SES may have contributed to their lower prevalence of body weight dissatisfaction. Finally, subsample sizes did not allow for an investigation of the individual obesity classes 1, 2 and 3 (i.e., BMI of 30–34.9, 35–40, and ≥ 40). Thus, specificities of severe obesity (classes 2–3)—e.g., in terms of disproportionally lowered health-related quality of life in men [38]—may have been overlooked. For instance, while among men the health care costs of severe obesity are known to be disproportionately higher than for other BMI groups (especially given high SES [39]), among women these costs tend to increase most when progressing from class 2 to class 3 obesity [40]. Thus, body weight dissatisfaction associated with specific (perceived or evaluated) need factors may be more prevalent in higher SES women with morbid obesity (class 3). At the same time, it can be asserted that the results for moderately obese women and men regarding the association between SES and the likelihood of being dissatisfied with one’s body weight reflected those for obese groups as a whole, with significant differences among men being even somewhat more pronounced, which, in a sense, makes the strategy to analyze moderate and severe obesity together seem rather conservative in terms of hypothesis testing. In other words, the finding that body weight dissatisfaction is more frequent in high socioeconomic groups among obese men and that there is a social gradient in body weight dissatisfaction in this group indicates that body weight satisfaction is not “merely” attributable to certain specificities (e.g., ill-health states) of the severely obese subgroup.

Bearing in mind the study’s limitations, its results statistically support previous evidence that body weight dissatisfaction is practically a normative discontent among women, not men (at least in European countries). A total of 48.3% of the women in this study were dissatisfied with their weight (men: 33%), a rate nearing the 50% which has been argued to constitute normative discontent [17]. While body weight dissatisfaction in bivariate (i.e., unstratified and unadjusted) analysis was negatively associated with women’s SES, no association was found within any of the three examined BMI categories. Thus, when compared to men, this bivariate association may be attributed to the relatively high rates of both low SES and obesity in women within the lowest SES group. By contrast, body weight dissatisfaction rates among men increased gradually with SES both overall and in the preobese and particularly the obese group. This suggests that, in this population of German adults, the premise that SES is unrelated to body size standards [23] may hold for women. Conversely, standards among overweight men in higher socioeconomic echelons may be more narrowly defined than those among their lower SES counterparts.

Nevertheless, the findings of this study should be tested against alternative interpretations since the study did not assess individual body image ideals. Using measures of abdominal obesity and muscularity, for example, which was beyond the scope of this analysis, may give hints as to whether the relatively low proportion of body weight dissatisfaction in lower SES obese men possibly reflects adherence to bodily standards other than fat-free leanness (i.e., less internalization of the thin ideal). In addition, psychosocial factors other than individual body ideals and (gender- and SES-specific) internalizations of the thin ideal may be operative [16,41]. First, importance of appearance—known to remain important throughout the lifespan of women [41] but not men [16]—may be less SES-specific among (pre-)obese women than men. Second, self-objectivation (defined as adopting an observer’s perspective of one’s physical self due to social construal of the body as an object to be evaluated) may be a mediating factor along a similar line of argument. Third, exposure to and perception of pressure from social environments, such as the media, may vary for both sexes in different SES groups. For instance, the number of magazines with a focus on men’s appearance and health has increased substantially over recent decades [42]. It is possible that this “innovation” has diffused (i.e., magazines have been read) more among men with a comparatively high SES given that in general “… individuals’ socioeconomic status is highly related to their degree of change agent contact” [43]. By contrast, publications focusing on women’s appearance and health have been around longer giving them more time to be diffused across the whole SES continuum.

Gender differences in the associations between body weight dissatisfaction and different SES indicators also merit discussion. First of all, in this study, the SES index and the individual SES indicators (educational level, income and occupational status) produced rather similar results in men, which of course is not always the case for other health or health-related outcomes [44]. The fact that the highest odds ratios pertained to the SES index compares well to previous results on perceived weight appropriateness [19]. A different pattern emerged for women in this study, however. Here, educational level stood out from the general trend of equality in body weight dissatisfaction across SES strata. Preobese women with a middle educational level and especially obese women with a high level of education had greater odds of being dissatisfied with their body weight than those in the respective lowest stratum. Although these results were somewhat inconsistent in the preobese group (middle not high educational level) and unstable in the obese group (large confidence interval), they may reflect gendered upbringing regarding body size standards. That is, given that education is a marker of childhood social environment resulting in differences in awareness among adults [44], shared socialization in the past may have affected both men and women. By contrast, current income and occupational status seem more relevant for men than women.

## Conclusions

Body weight dissatisfaction is a double-edged sword. It has been argued to be both a risk factor for obesity among young adults as well as a factor motivating weight loss in middle-aged or aged adults. The present finding that body weight dissatisfaction in German adults follows a dose–response gradient across socioeconomic strata in preobese and particularly in obese men (but not women) may point to obese men with a lower SES as a target group for obesity management. On the one hand, body weight satisfaction may partly explain why obesity is more frequent in groups with low socioeconomic status among men, while among women, SES seems unrelated to body size standards (even though the inverse social gradient in obesity is steeper than in men). On the other hand, combined with the fact that German men with low SES have particularly high odds of not exercising at all [45], health care and public health interventions should probably focus more on body image and, specifically, on body weight satisfaction in overweight men with relatively adverse socioeconomic backgrounds.

## Abbreviations

BMI: Body mass index; BWD: Body weight dissatisfaction; KORA: Kooperative Gesundheitsforschung in der Region Augsburg (Cooperative Health Research in the Region of Augsburg); S4: Survey 4 (of KORA); SES: Socioeconomic status; CI: Confidence interval; OR: Odds ratio.

## Competing interests

The authors declare that they have no competing of interest.

## Authors’ contributions

TvL managed data quality, performed the statistical analysis and drafted the manuscript. AM participated in the design of the study, managed data quality and helped draft the manuscript. The KORA Study Group consists of H.-E. Wichmann (speaker), R. Holle, J. John, T. Illig, C. Meisinger, A. Peters, and co-workers who are responsible for the design and conduct of the KORA studies. All authors read and approved the final manuscript.

## Pre-publication history

The pre-publication history for this paper can be accessed here:

http://www.biomedcentral.com/1471-2458/12/342/prepub
